# Management of multi-drug resistant *Helicobacter pylori* infection by supplementary, complementary and alternative medicine; a review 

**Published:** 2017

**Authors:** Nasim Rezaeimanesh, Nastaran Farzi, Samira Pirmanesh, Saeed Emami, Abbas Yadegar

**Affiliations:** 1 *Students Research Committee, * *Gastroenterology and Liver Diseases Research Center, Research Institute for Gastroenterology and Liver Diseases,* * Shahid Beheshti University of Medical Sciences, Tehran, Iran*; 2 *Foodborne and Waterborne Diseases Research Center, Research Institute for Gastroenterology and Liver Diseases, Shahid Beheshti University of Medical Sciences, Tehran, Iran*; 3 *Basic and Molecular Epidemiology of Gastrointestinal Disorders Research Center, Research Institute for Gastroenterology and Liver Diseases, Shahid Beheshti University of Medical Sciences, Tehran, Iran*; 4 *Gastroenterology and Liver Diseases Research Center, Research Institute for Gastroenterology and Liver Diseases, Shahid Beheshti University of Medical Sciences, Tehran, Iran*

**Keywords:** Helicobacter pylori, Drug resistance, Dietary supplementation, Anti-inflammatory, Eradication therapy

## Abstract

*Helicobacter pylori* is recognized as the most common bacterial pathogens colonizing the gastric epithelium of nearly half of the world’s population. This bacterium is the main etiological cause of gastroduodenal ulcers, and more importantly as the substantial risk factor for development of gastric cancer. The emergence and rapid increase in the prevalence of multi-drug resistant phenotypes have posed major pitfalls in effectiveness of various treatment regimens and eradication strategies against *H. pylori* infections. Several natural products and supplementary food components have been reported to have established anti-*H. pylori* activity. Herein, we review the application and efficacy of some specific natural products and foodstuffs such as milk, bee products (honey and propolis), fish oil, vitamins C and E, and also a nickel free-diet used as anti-*H. pylori* alternative treatment regimens.

## Introduction

*Helicobacter pylori* is a spiral bacterium that colonize in the human gastric epithelium. The bacterium causes different precancerous lesions like gastritis, atrophy, intestinal metaplasia and dysplasia, and is the strongest known risk factor for gastric cancer (GC) (1-3). *H. pylori* infects more than half of the people globally, and the prevalence of *H. pylori* infection is highly variable across different countries; for example, high prevalence is observed in developing countries (~80%) in comparison to developed countries with about 30-50% of the population (4). The bacterium usually is acquired in childhood and can persist for lifetime in the host stomach. *H. pylori* pathogenesis is mediated by a complex interplay between various bacterial virulence factors, host genetic predisposition, and environmental factors (5-7). *H.* *pylori* is also known as one of the most genetically diverse bacterial species that presents various virulence genotypes responsible for different gastric diseases (8-10). The emergence and more importantly the increasing prevalence of multi-drug resistant strains of *H. pylori* has led to reduced success in different treatment regimens (11-17). Several natural products and supplementary nutrients have been reported to have established antimicrobial activity against *H. pylori *infection (Table 1). Here, we tried to have a short overview on the application and efficacy of some natural products and supplementary compounds used as anti-*H. pylori* alternative treatments.

**Dairy, bee products and fish oil, Vitamins, Nickel free-diet**

**Milk**

Milk, especially the human milk, has long been identified as one of the natural products encompassing high nutritional values as well as antimicrobial effects against a variety of infections. Before the discovery of antibiotics, a non-specific therapy named protein therapy, used the antibacterial properties of milk protein contents (18). Moreover, nutrient fortification of human milk with medium-chain triglycerides (MCT) and iron has been also applied as an acceptable supplement therapy for feeding preterm infants (19).

Lactoferrin is a multifunctional iron-binding glycoprotein with potent antibacterial and immunomodulatory properties against several bacterial pathogens (20, 21). It is released in the human colostrums with highest concentration in comparison with human milk and cow milk (20). It is also present in saliva, tears, seminal fluid and immune cells like neutrophils (20, 22). Lactoferrin has been shown to have inhibitory effects on growth of a number of bacterial pathogens including *Streptococcus mutans*,* Streptococcus penumoniae*, *Haemophilus influenza*, *Neisseria meningitidis*, *Escherichia coli *and* H. pylori* (21, 23). The antimicrobial activity of lactoferrin is well studied and is likely attributed to its high affinity for iron, functioning as an iron chelator that sequesters iron elements from bacterial access (22, 24).

There are some in vivo studies showing that lactoferrin can improve *H. pylori* eradication rate in humans and mouse models (25-28). In another in vitro study, Akedo et al. showed anti-*H. pylori* property of cow’s milk (29). Moreover, in a survey on 482 children aged 0-12-years-old, Okuda and et al. assessed the relation between breast feeding and *H. pylori *infection, and proposed breast feeding can be a natural way to protect children from infection due to anti-adhesive property of lactoferrin, sialyllactose and oligosaccharides present in breast milk (30). In 2013, a review study suggested that fermented milk-based probiotic preparations and bovine lactoferrin can be effective for *H. pylori* eradication (31).

**Honey**

In the traditional medicine, honey was used as an anti-microbial substance for treatment of infectious diseases, and also gastrointestinal disorders like dyspepsia, gastritis, peptic ulcer disease, and liver disease (27-29). Additionally, it is reported that honey can accelerate wound healing process and was used to dress wounds and burns (32). The antibacterial property of honey varies due to its color and floral sources. Red honeys have shown more potent antibacterial properties than white honeys, which this property depends on different phenolic acid contents of various kinds of honeys (33, 34). The bactericidal and bacteriostatic potency of honey is broad-spectrum, and is effective against several bacterial agents such as *Staphylococcus aureus*, *Pseudomonas aeruginosa*, *E. coli *and *Streptococcus pyogenes *(34). It has been proposed that antibacterial activity of honey is mainly due to its high osmotic and acidity, hydrogen peroxide production, flavonoids and over-mentioned phenolic acids content (35-38).

Some studies revealed that high osmotic and high acidity effects of honey can inhibit the urease activity of *H. pylori* (37, 38). Abdel-Latif and colleagues investigated the molecular mechanisms by which natural honey may inhibit *H. pylori* infection. They reported that Manuka honey can inhibit *H. pylori* by suppression of *H. pylori* induced NF-kB and AP-1 activation, and down regulation of COX-2 expression in gastric epithelial cells (35). Another in vivo study in Bulgaria showed the anti-*H. pylori* effect of honey via its high osmotic and acidity effect (36).

**Propolis**

Propolis is a resinous bee product that contains plant resin, bee enzymes and wax (39, 40). It is reported to be an antioxidant, antibacterial, antifungal, anti-inflammatory, antiproliferative and antidiabetic substance. The natural composition of propolis varies due to its original floral sources, and contains different amounts of phenolic compounds (39-41). The anti-*H. pylori* properties of propolis may be due to its phenolic substances such as flavonoids, phenolic acids and their esters like caffeic acid phenethyl ester (CAPE) and chrysin (40-42). Baltas and colleagues studied the anti-*H. pylori* effect of 15 ethanol extracts of propolis and reported that all extracts inhibited *H. pylori* J99 strain by urease inhibition (41). In 2013, Cui et al. assayed different propolis phenolic compounds for *H. pylori* peptide deformylase (HpPDF) inhibition, which is necessary for *H. pylori* perpetuity and persistence (40).

**Table 1:** Specific food and natural products with potential anti-*H. pylori* activities

Food types	Active components	Putative anti-*H. pylori* properties	Testing methods	Year/country	Ref.
Milk	Lactoferrin, sialyllactose, oligosaccharides	Inhibition of *H. pylori *attachment	*H. pylori* stool antigen assay (HpSA)	2001/Japan	30
	Lactoferrin adsorbed into biomimetic hydroxyapatite nanocrystals	Iron chelating and sequestration	Inhibition zone assay and the bacterial load were measured in orally *H. pylori*-infected BALB/c mice using SYBR Green I quantitative real-time PCR assay	Italy/2016	67
	Bovine milk glycoproteins and glycoconjugates and lactoferrin	Iron deprivation, decreasing gastric colonization of* H. pylori* and inflammation score	Growth inhibition assay, haemagglutination inhibition assay and adherence assay	Sweden/2001	28
Honey	Hydrogen peroxide and phytochemicals (flavonoids and phenolic acids)	High osmotic effect, pH (high acidity)	^13^C UBT	Bulgaria/2015	36
	Flavonoids and phenolic acids	Inhibition of bacterial urease activity	Urease activity assay by spectrophotometry	South Africa/2014	38
	Hydrogen peroxide and phytochemicals (flavonoids and phenolic acids)	High osmotic effect, pH (high acidity)	Hole plate diffusion method and microbroth dilution method	Cameroon/ 2013	37
	Flavanoids, phenolic acids, vitamins, trace elements, amino acids, proteins, certain enzymes including glucose oxidase, invertase and catalase	Inhibition of *H. pylori*-induced NF-kB and AP-1 activation and downregulation of COX-2 expression, growth inhibition	Electrophoretic mobility shift assay (EMSA), cell viability assay and cytotoxicity assay	Egypt/2016	35
Propolis	Flavonoids, Phenolic compounds, Caffeic acid phenethyl ester and chrysin	Urease inhibition	Agar-well diffusion method and urease inhibition assay	Turkey/2016	41
	Phenolic compounds, Caffeic acid phenethyl ester	Inhibition of* H. pylori* peptide deformylase	Enzymatic activity of *Hp*PDF was evaluated using a FDH coupled assay	China/2013	40
Fish oil	EPA, DHA, Omega 3 fatty acids	Direct inhibition of bacteria, antiadhesive activity, anti-inflammatory effect	Agar diffusion test (Kirby-Bauer method)	Italy/1999	52
Vitamin C	Ascorbic acid	*H. pylori* growth inhibition, urease inhibition, antioxidant effects	Urease test and histological examination (Giemsa staining)	Poland/1998	61
Vitamins C and E	Ascorbic acid, tocopherols and tocotrienols	Inhibition of *H. pylori *colonization, antioxidant effects	Rapid urease test, histopathological evaluation and UBT	Turkey/2015	62
	Ascorbic acid, tocopherols and tocotrienols	Antioxidant effects	Histologic examination, rapid urease test, ^14^C-urea breath test, HpSA and Measurement of Total Antioxidant Capacity (TAC)	Turkey/2009	56

Abbreviations: UBT, urease breath test; *Hp*PDF, *H. pylori* peptide deformylase; FDH, formate dehydrogenase; PCR, Polymerase chain reaction; PH, potential of hydrogen; NF-κB, nuclear factor kappa-light-chain-enhancer of activated B cells; AP-1, Activator protein 1; COX-2, Prostaglandin-endoperoxide synthase 2 (cyclooxygenase-2); EPA, Eicosapentaenoic acid; DHA, docosahexaenoic acid

**Fish oil**

From many years ago, essential oils were among the most useful components in traditional medicine around the world and their activity against *H. pylori* have been delineated (43, 44). Fish oil (Eicosapen) includes 33.5% omega-3-fatty acids with a variety of immunomodulating effects, which has bacteriostatic effect on *H. pylori *(45-49). Moreover, it has been reported that omega-3-fatty acids declined the secretion of gastric acid in healthy volunteers (50). It has been proposed that the inhibitory effects of fish oil on *H. pylori* may be due to: 1) direct inhibition or killing the bacteria (49), 2) inhibition of bacterial adhesion to gastric epithelium, and 3) inhibition of the *H. pylori*-induced inflammatory pathways (51, 52).

**Vitamins**

Vitamin C, an acidic molecule, is one of the most important component of living tissues. Two forms of vitamin C including: AA (ascorbic acid) and DHA (dehydroascorbic acid), the reduced and oxidized form, respectively, which can convert to each other. Inside the cell, DHA is immediately converted to AA in presence of glutathione or other thiols as electron donors via the specific enzyme systems like DHA reductase, glutaredoxins and protein disulfide isomerase (53, 54). Unfortunately, stability of AA and DHA are low and have a rapid wild irreversible hydrolysis particularly at a pH > 4 (55). Vitamins C and E have been studied to show their antioxidant effect for eradication of *H. pylori *infection (56, 57). It seems vitamins C and E break the microenvironment created by *H. pylori *or directly inhibit bacteria. Additionally, the detriment of antioxidants on colonization and proliferation of *H. pylori *have been shown (58-60).

In a study by Sezikli et al., they showed that under the oxidative stress vitamins C and E were effective in eradication of *H. pylori* infection (56). In another work, administration of high dose vitamin C treatment had inhibitory effects on *H. pylori* growth (61). In a study by Demirci and coworkers, the effect of vitamins C and E supplementation along with triple and quadruple eradication regimens was assessed using 400 *H. pylori* infected patients. They showed that *H. pylori* eradication rate was 56% for smokers and 94% for non-smokers. The success rate of *H. pylori* eradication for smokers was lower than non- smokers (62). Zojaji et.al, also reported that addition of vitamin C to *H. pylori* treatment regimen including amoxicillin, metronidazole, and bismuth increased the eradication rate among the infected patients (63).

**Nickel free-diet**

Nickel is a metallic element that is widely found in almost all kinds of diets (64). It is abundant in fruits and vegetables like apricots, figs, pears, plums, raisins, pineapples, cabbage, onions, beans, lentils, potatoes, peas, tomatoes, spinach, cauliflower, asparagus, corn and margarine. This element is also present in different kinds of nuts including almonds, peanuts, walnuts, hazelnuts, and cocoa as well as some sea foods like lobster, mussels, oysters and plaice (65, 66). However, it seems that nickel is not essential for humans, but it is necessary for *H.* *pylori *colonization because of its important role in activation of *H. pylori* urease and hydrogenase enzymes. So, there is no competition between *H. pylori* and human body for nickel access (64). According to these facts, Campanale and colleagues in 2014 designed a pilot study to investigate the effect of a nickel free-diet on the eradication of *H. pylori *infection. In their in vivo study, 52 participants with *H. pylori* infection were divided into two groups: standard triple therapy and standard triple therapy with nickel free-diet. In the second group, the participants were prohibited to consume foods with high quantity of nickel for 4 weeks. They found that addition of nickel free-diet to standard triple therapy can significantly promote *H. pylori* eradication rate (64).

## Discussion

Currently, the treatment of all symptomatic *H. pylori*-infected patients is less probable, and could rapidly increase the emergence and prevalence of multi-drug resistant strains in the community. Moreover, despite the availability of several therapeutic strategies for *H. pylori-*induced gastric diseases, the bacterial eradication is very challenging and none of the treatment regimens appear to be ideal. Therefore, the application of relatively low-cost natural products and foodstuffs with established anti-*H. pylori *activity seems to be promising as alternative medicine and adjuvant therapy to manage the infections caused by antibiotic-resistant *H. pylori* strains. However, it is very important to evaluate the antibacterial effectiveness of different natural products and food components by both *in vitro *and *in vivo *experiments, especially in the clinical trials, to propose a potentially effective diet-based treatment regimen (68). Finally, further studies are needed to explore novel, local and natural therapeutics to be co-administrated with conventional antimicrobial agents as adjunctive therapy against *H. pylori* infections.
